# Abscisic acid interplays with PPARγ receptors and ameliorates diabetes-induced cognitive deficits in rats 

**Published:** 2021

**Authors:** Razieh Kooshki, Akbar Anaeigoudari, Mehdi Abbasnejad, Khadijeh Askari-Zahabi, Saeed Esmaeili-Mahani

**Affiliations:** 1 *Department of Biology, Faculty of Sciences, Lorestan University, Khorramabad, Iran*; 2 *Department of Physiology, School of Medicine, Jiroft University of Medical Sciences, Jiroft, Iran*; 3 *Department of Biology, Faculty of Sciences, Shahid Bahonar University of Kerman, Kerman, Iran*; 4 *Neuroscience Research Center, Institute of Neuropharmacology, Kerman University of Medical Sciences, Kerman, Iran*

**Keywords:** Diabetes mellitus, Streptozotocin, Abscisic acid, Long term potentiation, Learning and memory, Rats

## Abstract

**Objective:**

This study intended to evaluate if central administration of abscisic acid (ABA) alone or in combination with GW9662, a peroxisome proliferator–activated receptor γ (PPAR-γ) antagonist, could modulate learning and memory as well as hippocampal synaptic plasticity in a rat model of streptozotocin (STZ)–induced diabetes.

**Materials and Methods:**

Intraperitoneal injection of STZ (65 mg/kg) was used to induce diabetes. Diabetic rats were than treated with intracerebroventricular (i.c.v.) administration of ABA (10, 15 and 20 µg/rat), GW9662 (3 µg/rat) or GW9662 (3 µg/rat) plus ABA (20 µg/rat). Animals’ spatial and passive avoidance learning and memory performances were assessed by Morris water maze (MWM) and shuttle box tasks, respectively. Further, *in vivo* electrophysiological field recordings were assessed in the CA1 region.

**Results:**

STZ diabetic rats showed diminished learning and memory in both MWM and shuttle box tasks. The STZ-induced memory deficits were attenuated by central infusion of ABA (10 and 20 µg/rat). Besides, STZ injection impaired long-term potentiation induction in CA1 neurons that was attenuated by ABA at 20 μg/rat. Central administration of GW9662 (3 µg/rat) alone did not modify STZ-induced spatial and passive avoidance learning and memory performances of rats. Further, GW9662 prevented ABA capacity to restore learning and memory in behavioral and electrophysiology trials.

**Conclusion:**

Altogether, ABA ameliorates cognitive deficits in rats via activation of PPAR-γ receptor in diabetic rats.

## Introduction

Abscisic acid (ABA) is a sesquiterpenoid phytohormone necessary for regulating plant responses to abiotic stresses, seed growth, and sugar sensing (Raghavendra et al., 2010 ▶). It is also produced by metazoans, from sponges up to mammals (Zocchi et al., 2017 ▶). ABA pathway is highly conserved across species and it has been known as a potent agonist for lanthionine synthetase C-like 2 receptor and peroxisome proliferator-activated receptors family member (PPARs) (Kline et al., 2010 ▶). ABA stimulates Ca^2+^ release by activation of downstream targets including phospholipase C / protein kinase C (PLC-PKC) cascade and adenylate cyclase cAMP-dependent protein kinase A (PKA) pathway (Hauser et al., 2017 ▶). 

In addition to its vital functions in plants, ABA exerts critical roles in controlling physiological functions in animals. In particular, ABA has been considered a novel nutritional therapeutic for controlling glucose metabolism and it could regulate glucose load in both genetically and dietary-induced obese mice (Sturla et al., 2017 ▶; Zocchi et al., 2017 ▶). Furthermore, ABA production and secretion is increased subsequent to increased plasma glucose levels (Lievens et al., 2017 ▶). In addition, ABA and insulin have an equal potential of stimulating glucose uptake in murine adipocytes and rats myoblasts *in vitro* (Zocchi et al., 2017 ▶).

The involvement of ABA in memory and cognitive processes has been also documented. ABA increased spatial learning and memory performance when it was administered orally or centrally in rats (Naderi et al., 2017 ▶; Qi et al., 2015 ▶). Besides, ABA injection into the lateral ventricles diminished cognitive deficiency in an animal model of Alzheimer's disease. Interestingly, ABA effect was repressed by inactivation of PPAR β/δ receptor (Khorasani et al., 2019 ▶). 

Diabetes is a multifactorial disease that is usually combined with long lasting dysfunction and failure of multiple organ systems in body (Giri et al., 2018). Diabetes mellitus (DM) type 1 is induced by destruction of insulin-secreting pancreatic beta cells. Insulin is critically involved in regulation of glucose metabolism and metabolic processes in the brain and peripherals tissues (Gerozissis, 2008 ▶; Lotfy et al., 2017 ▶).

A number of rodent models of diabetes has been designed to predict anti-diabetic drugs efficacy. In particular, streptozotocin (STZ) is frequently used for inducing DM in rodents. STZ is extremely injurious to the beta cells in the pancreas and could be administered at different concentrations and via various routes to induce severe or mild diabetes (Arora et al., 2009 ▶; Furman, 2015 ▶).

Diabetes and subsequent hyperglycemia raise several neurological complications. In particular, clinical and preclinical studies have shown the destructive effects of DM on cognitive functions (Ahmed et al., 2019 ▶; Popoviç et al., 2001 ▶). It has been indicated that STZ-induced diabetes disrupts neurogenesis and synaptic plasticity in rats’ hippocampal pathways. It might be mediated by disruption of neural pathways and deficiency in immunity and metabolism processes (Shonesy et al., 2012 ▶; Xu et al., 2014 ▶). 

Transcription factors PPARs have been identified as α, β/δ, and γ subtypes. The PPARs were shown to play vital roles in glucose hemostasis and lipid metabolism (Kota et al., 2005 ▶). In particular, PPARγ-targeting  anti-diabetic drugs like thiazolidinediones (TZDs) are able to decrease hyperglycemia and insulin resistance in diabetes type 2 (Yasmin and Jayaprakash, 2017 ▶). Besides, pharmacological stimulation of PPAR-*γ* receptors was shown to ameliorate metabolic challenges and memory impairments in STZ-diabetic mice (Liu et al., 2013 ▶). In addition, a PPARγ agonist has been shown to ameliorate the amyloid *beta*-mediated impairment of synaptic transmission and plasticity in the CA1 area (Costello et al., 2005 ▶). Besides, PPAR-γ activation could restore synaptic plasticity and dendritic spine densities deficits in obese insulin-resistant rats (Sripetchwandee et al., 2014 ▶).

The imperative roles for ABA in learning and memory and diabetes processes have been indicated in previous studies. However, ABA ability to improve cognitive performance in diabetic rats has not yet been evaluated. Therefore, this study was designed to evaluate if central administration of ABA could modulate passive avoidance and spatial learning and memory as well as hippocampal synaptic plasticity in diabetic rats. Further, because of data indicating ABA interaction with PPAR signaling, GW9662, a PPAR-γ antagonist, was used to assess possible association of ABA with PPAR-γ in modulation of cognitive performances in diabetic rats. 

## Materials and Methods

**Animals**

Male Wistar rats (weighing 230-250 g, 13 weeks old) were used. The animals were preserved in a temperature-restrained area (23±1°C) under a regular light (12 hr)/ dark (12 hr) cycle with *ad libitum* access to food and water. The experimental procedures were legalized by the ethical committee of Jiroft University of Medical Sciences, Jiroft, Iran (IR.JWU.REC.1397.42).

**Surgery**

The animals were anesthetized by injections of ketamine and xylazine (50 and 5 mg/kg, respectively) (Struck et al., 2011 ▶) and placed in a stereotaxic apparatus. The guide cannulas (23 gauge) were fixed in the lateral ventricles according to the atlas of Paxinos and Watson (Paxinos and Watson, 1998 ▶) (1.6 mm posterior to the bregma±0.8 lateral from the midline and at a depth of 3.4 mm from the pial surface). Then, the rats were reserved in individual cages and allowed one-week recovery from surgery. After completion the tests, the rats were decapitated and brain cannula inserting was done for each rat by injection of 0.5 μl of methylene blue via the cannula. In case of misplaced location, the rat’s data was excluded from analysis. 

**Drugs and microinjection**

The STZ was dissolved in normal saline and injected intraperitonealy (i.p.) using an insulin syringe (30 gauges*)*. (±)-cis, trans-abscisic acid and GW9662 (both Sigma-Aldrich, USA) were dissolved in dimethyl sulfoxide (DMSO) and then, diluted using *artificial cerebrospinal fluid* (aCSF). GW9662, ABA and drugs vehicles were administered into lateral ventricles at a rate of (1 μl/min/rat) through an internal cannula (27-gauge) attached to a 1 μl Hamilton syringe via a polyethylene tube.

**Diabetes induction**

Diabetes was induced by a single i.p. injection of STZ (65 mg/kg) and established by the incidence of obvious hyperglycemia, polyphagia, and weight loss. In addition, one week after the induction of diabetes, blood samples were collected and serum glucose concentrations were evaluated using a glucose kit (Zistshimi, Iran), using the enzymatic glucose oxidase method.  The rats with serum glucose levels upper than 250 mg/dl were considered diabetic for use in subsequent experiments.

**Experimental groups**

The rats were randomly allocated to seven groups (n=6): sham, STZ, STZ+ ABA (10, 15, and 20 µg/rat), STZ+ GW9662 (3 µg/rat), and STZ+ GW9662+ABA. Animals in the sham group were injected i.p. with STZ vehicle normal saline and i.c.v. with DMSO as a vehicle for ABA and GW9662; the STZ group was injected i.p. with STZ; three STZ+ABA- treated groups were microinjected with ABA at different doses (10, 15, and 20 µg/rat) after STZ administration; STZ+ GW9662 group was microinjected with GW9662 (3 µg/rat) as a PPAR-γ antagonist subsequent to STZ administration and STZ+ GW9662+ABA group was injected with STZ and then treated by GW9662 (3 µg /rat) 15 min prior to ABA administration (20 µg/rat /i.c.v.). In all the experimental groups, central administrations of drugs, ABA or GW9662, were performed one week after STZ administration and diabetes verification.

**Learning and memory assessment**

**Spatial learning and memory**

A black circular swimming pool (60 cm high) was filled with water (22±1°C) to a depth of 25 cm. The Morris water maze (MWM) pool was separated conceptually into four equal zones and a dark circular iron platform was situated 2 cm under the water surface in the middle of one of the zones approximately 25 cm from the wall of the pool. The optical extra-maze cues were hung on the walls of the experiment room. The experiments comprised of acquisition and probe (memory) tests. Acquisition phase was consisted of three blocks with four trials per block. In each trial, a rat was permitted to swim the swimming pool for 60 sec to find the hidden platform submerged under the water surface. If a rat did not find the platform within one minute, the experiment was finished and a maximum score of one minute was considered. The escape latency time and the swimming distance travelled, were recorded by computerized Any-maze video tracking system (Stoelting Co., USA). On the next day, in the probe trial, the platform was omitted and the animals were positioned in the zone opposed to the target zone and permitted to swim for one minute. Throughout the trial, the total time that rats disbursed in the target zone, was regarded as an indicative of spatial memory capacity.

**Passive avoidance test**

The maze was made of two equal illuminated and dark cavities associated to each other by a Plexiglass gateway. At the habituation phase, the rats were located in the light zone of maze and indorsed to ambulate without restrictions between the two cavities of the maze for 5 min. In the learning course, each rat was located in the light cavity and then the door was opened and the rat was allowed to go to the dark cavity. Then, the guillotine gate was closed and an electric shock (0.5 mA for 4 sec) was directed towards the animals and then the animal was returned to the cage. Five minutes later, the same trial was accomplished. If the rat did not move in the dark cavity throughout the 300 sec, the learning phase was considered effective. The number of learning trials to get efficient learning, was recorded. On the next day, in retention trial (memory test), the rats were positioned in the illuminated chamber and allowed to cross into the black cavity. The time needed to arrive in the dark cavity, step-through latency (STL) and total time spent in the dark chamber (TDC) were recorded as contextual learning index. 

**Field potential recording**

The animals were anesthetized by urethane (1.2 g/kg/i.p.) and placed in a rat stereotaxic apparatus. Under sterile conditions*, *a recording stainless-steel electrode (125 µm in diameter) was inserted in the stratum radiatum of the right hippocampus CA1 (4 mm: anterior to bregma , and 3 mm: lateral to bregma) and a 125 µm bipolar stimulating electrode was inserted into the ipsilateral Schaffer collateral pathway (3 mm anterior and 3.5 mm lateral to bregma). The *electrodes were lowered slowly* in the brain to get a suitable response. For baseline synaptic potency, proliferative stimulus strength with constant current, was considered input and the field excitatory postsynaptic potential (fEPSPs) was verified as output. The stimulus intensity with 50% of the maximum amplitude of the peak potential response, was considered baseline synaptic response. The signals were amplified 100 times and filtered between 1 to 3000 Hz. The baseline response was recorded for 30 min. Then, *LTP* was *induced* by a train of high frequency stimulation (HFS) (trains of 10 pulses at 400 Hz repeated at intervals of 7 sec, for 70 sec). After the HFS, the response was evaluated for 120 min and the magnitudes of fEPSP slope were specified as the average of 10 consecutive traces at each time point.

**Statistical analysis**

All of values are expressed as mean±SEM. The normality of data was evaluated with Kolmogorov-Smirnov test. The data of acquisition trials in MWM test and LTP time points values were analyzed by parametric repeated-measures analysis of variance (ANOVA). In addition, one-way ANOVA was done to assess differences in the probe trial test. *Post hoc* analysis was done using the Tukey test. Further, the data of shuttle box test did not show normal distributions*. *So, the data were analyzed using Kruskal-Wallis test. A p value less than 0.05 was considered significant. 

## Results

**Spatial learning and memory test**

**Figure 1:**
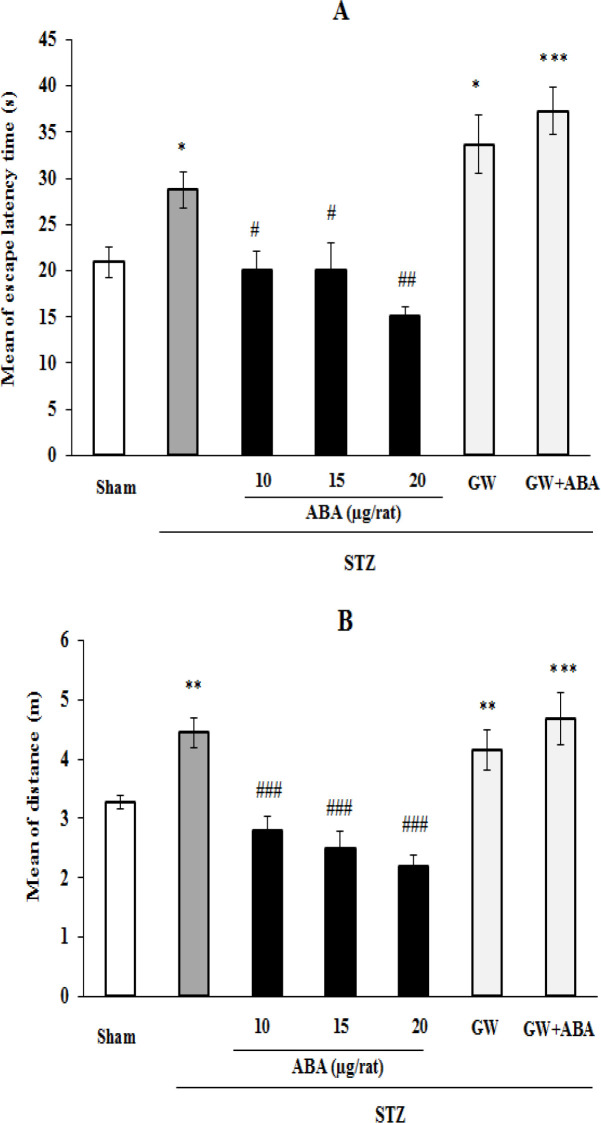
Effects of central administration of ABA (10, 15 and 20 μg/rat), GW9662 (3 μg/rat), and GW9662 (3 μg/rat) plus ABA (20 μg/rat) on (A) latency and (B) distance travelled in acquisition blocks in STZ-diabetic rats. Data are expressed as mean±SEM. **p＜0.001, **p＜0.01, and *p＜0.05 versus sham, ^###^p＜0.001, ^##^p＜0.01, and ^#^p＜0.05 versus STZ group. STZ: streptozotocin, GW: GW9662 and ABA: abscisic acid

There was a significant difference in the mean escape latency to find the platform among the groups [F (6,167)=20.701, p=0.0001]. The STZ-treated rats showed a longer latency to find the platform in comparison with the sham (p*<*0.05) and ABA-treated groups (at 10 and 15 μg/rat, p*<*0.05 and at 20 μg/rat, p*<*0.01). However, GW9662 (3 μg/rat, i.c.v.) infusion repressed ABA (20 μg/rat)-decreased latency to find the hidden platform in STZ-diabetic rats (Figure 1A). 

In addition, the groups showed changes in a travelled distance to get the hidden platform [F (6,167)=14.614, p=0.001]. Treatment with STZ significantly increased mean travelled distance to reach the hidden platform as compared to the sham-treated rats (p<0.01). Moreover, microinjection of ABA at all tested doses, decreased STZ-increased travelled distance to catch the hidden platform (p<0.001; Figure 1B). Moreover, injection of PPAR antagonist GW9662 (3 μg/rat) did not modify STZ-increased path traveled for finding the hidden platform. However, it was able to repress ABA (20 μg/rat)-decreased path travelled to find the platform in STZ-diabetic rats (Figure 1B). 

**Figure 2:**
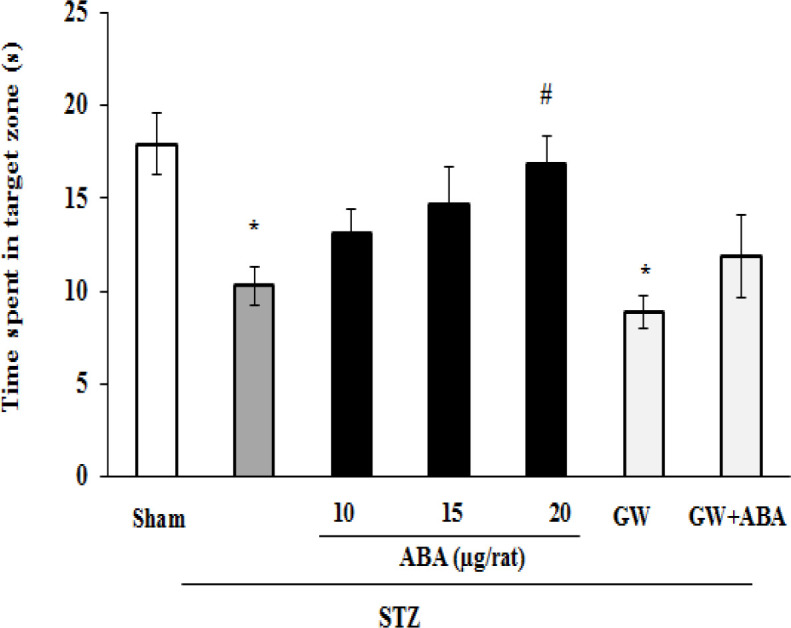
Effects of central administration of ABA (10, 15 and 20 µg/rat), GW9662 (3 μg/rat), and GW9662 (3 μg/rat) plus ABA (20 μg/rat) on time spent in the target zone in STZ-diabetic rats. Data are expressed as mean±SEM. *p＜0.05 versus sham; and ^#^p＜0.05 versus STZ. STZ: streptozotocin, GW: GW9662 and ABA: abscisic acid

In the probe trial, STZ-treated rats spent less time in target zone in comparison to the sham group (p<0.05). However, the STZ effect was suppressed by treatment with ABA (20 μg/rat) (p<0.05). Furthermore, central infusion of GW9662 (3 μg/rat) alone had no effect, however, it could prevent ABA-induced increased time spent in the target zone in STZ-diabetic rats (Figure 2). 

**Passive avoidance test**

**Figure 3:**
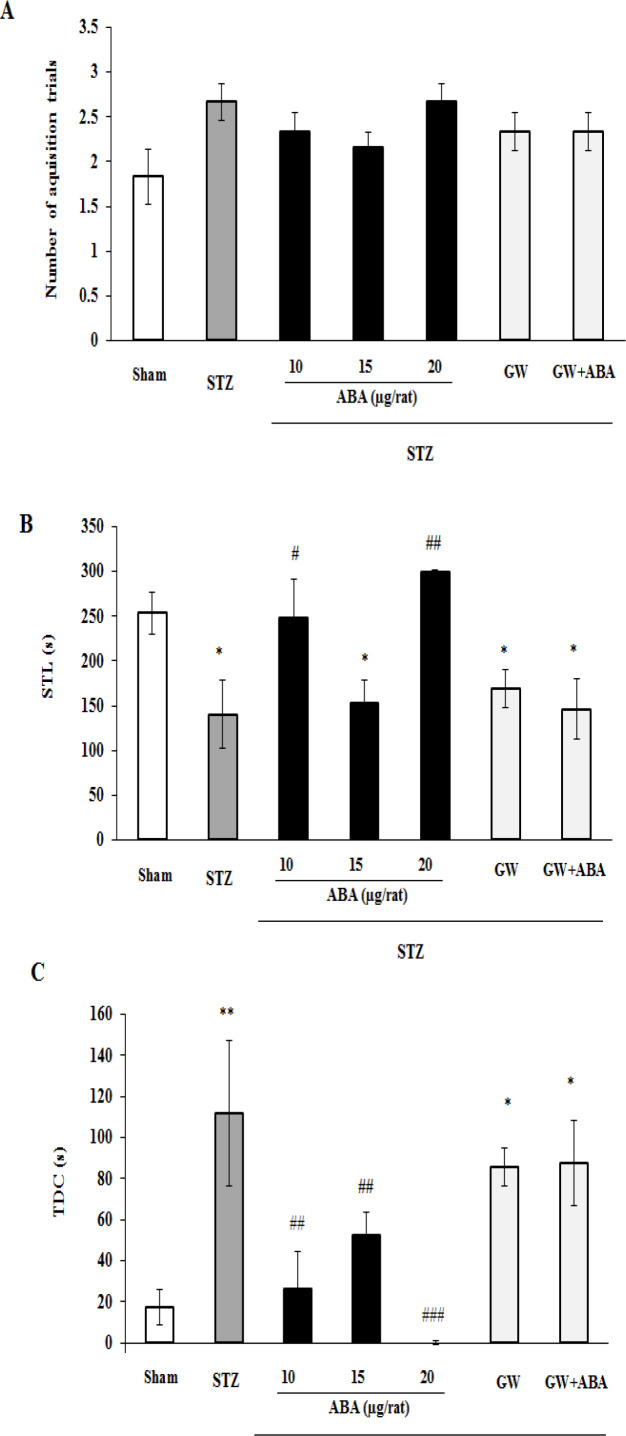
Effects of central administration of ABA (10, 15 and 20 µg/rat), GW9662 (3 μg/rat), and GW9662 (3 μg/rat) plus ABA (20 μg/rat) on (A) the number of acquisition trials, (B) step-through latency (STL) and (C) total time spent in the dark chamber (TDC) in STZ-diabetic rats in shuttle box test. Data are expressed as mean ± SEM. **p＜0.01 and *p＜0.05 versus sham, ###p＜0.001, ##p＜0.01 and #p＜0.05 versus STZ group. STZ: streptozotocin, ABA: abscisic acid and GW: GW9662

The groups did not show major differences in the number of acquisition trials to get successful learning [p=0.217]. However, STL [p=0.001] and TDC [p=0.001] were significantly different among the groups. Mann Whitney-U *post hoc* indicated that STZ administration could significantly reduce STL (p<0.05) and increase TDC (p<0.01) in comparison to sham treatment. However, ABA at 10 µg (p<0.05) and 20 µg (p<0.01) attenuated STZ effects. Moreover, STZ-increased TDC was significantly suppressed by ABA at 10 and 15 µg (both p<0.01) as well as 20 µg (p<0.001). Furthermore, the results showed that inactivation of PPAR-γ receptor by GW9662, had no effect on acquisition trials for learning in STZ-treated rats (Figure 3A). However, GW9662 was able to inhibit ABA aptitude to increase STL and decrease TDC in STZ-diabetics rats (Figures 3B and 3C). 

**Field potential recording**

**Figure 4:**
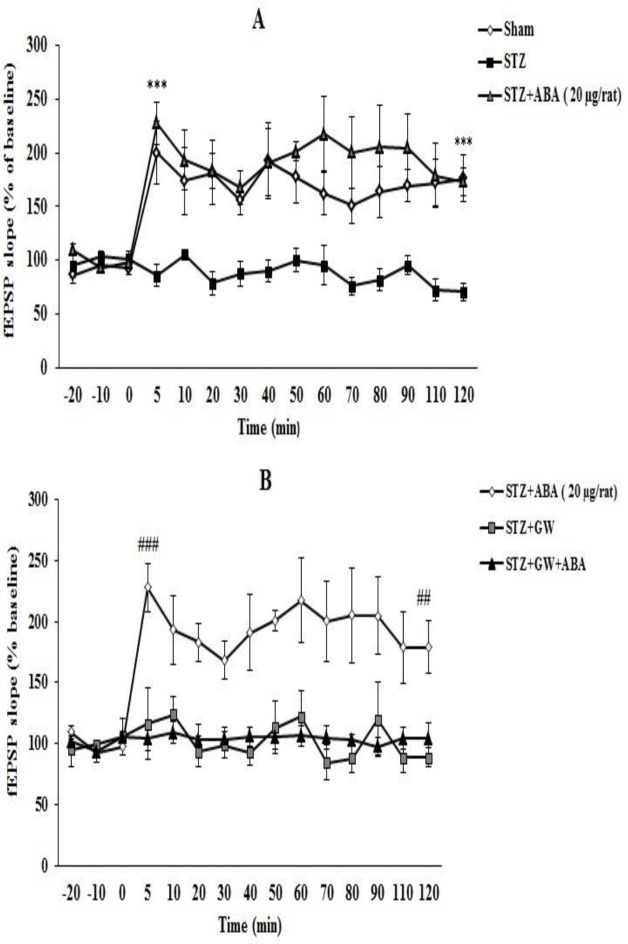
Comparison of differences in the induction of LTP in the CA1 area of the hippocampus amongst different experimental groups. Data are expressed as mean percentage change from baseline responses± SEM. ^***^p< 0.001 versus STZ group, ^###^p< 0.001, ^##^p< 0.05 versus STZ+GW and STZ+GW+ABA groups. STZ: streptozotocin, ABA: abscisic acid, and GW: GW9662

As shown in Figure 4A, E-LTP was evoked in the sham group that continued over 2 hr after HFS. Induction of LTP was impaired in STZ diabetic rats. Administration of ABA (20 µg/rat) improved the induction and maintenance of LTP in STZ- treated rats (p<0.001). Moreover, LTP was affected in groups of rats treated by GW9662 (3 μg/rat) and GW9662 plus ABA (20 µg/rat) after STZ administration (Figure 4 B). 

## Discussion

In this study, central infusion of ABA, in a dose-dependent manner, ameliorated spatial and passive avoidance learning and memory deficits in STZ-induced diabetic rats. In the MWM test, ABA, at all doses, inhibited STZ-induced spatial learning impairment. Besides, diabetic rats that received 10 and 20 µg/rat of ABA, showed better memory retention in passive avoidance test. Furthermore, ABA (20 µg) could repair hippocampal synaptic plasticity deficiency induced by STZ. However, the ABA effects in both behavioral and electrophysiology experiments, were prevented by PPARγ receptor antagonist GW9662. 

Here, as shown in Figure 3B, on step-through latency time, ABA was effective at low (10 µg) and high (20 µg) dose but not at moderate dose (15 µg). It has been demonstrated that drug efficacy does not always increase sigmoidally with concentration. Unlike standard sigmoidal curves, bell-shaped concentration–response curves suggest more complex biological effects, multiple binding sites or multiple targets. It means that U-shaped curves are resulted from a single drug having more than one mechanism of action (Owen et al., 2014 ▶). Molecular studies have shown that ABA can activate different types of receptors and multiple intracellular signaling molecules. Therefore, such multi-target or multi-functional agent may produce a non-sigmoidal dose-response curve for some of its actions. However, further experiments are necessary to determine the pharmacodynamics of ABA in animals, and to elucidate the causality of non-sigmoidal dose-response effect of ABA.

In the present study, ABA protected against STZ-induced cognitive dysfunction. In support, it was reported that central or peripheral infusion of ABA is able to increase learning and memory performances in rats (Naderi et al., 2017 ▶; Qi et al., 2015 ▶). Moreover, in a recent study, administration of ABA into the lateral ventricles attenuated passive prevention and spatial learning and memory deficits in a rat model of STZ- induced Alzheimer's disease (Khorasani et al., 2019 ▶). 

ABA intracellular signaling pathway is not yet adequately defined in animals, however, it is considered an agonist for PPAR members’ family and LANCL2 receptors. In this study, PPARγ receptors antagonist prevent ABA ability to increase learning and memory performances in diabetic rats. In line with our data, it was indicated that PPAR β/δ receptors blockade prevent ABA moderating effect on thermal nociception and formalin-induced inflammation in rats (Mollashahi et al., 2018 ▶). In addition, the defensive effect of ABA against STZ-induced AD was suppressed in response to PPAR receptors inactivation, in rats (Khorasani et al., 2019 ▶). ABA activates several signaling molecules that have pivotal roles in learning and memory processing such as cAMP/cAMP-dependent PKA, MAPK, PKC and phosphatidylinositol-3-kinase (PI3K). It was reported that PKC or PI3K inhibitors are able to block ABA pro-cognitive and anxiolytics effects in rats (Naderi et al., 2019 ▶). There is an association between PPARγ receptors activity and increased intracellular cAMP levels and PKA expression (Chen et al., 2013 ▶; Singh et al., 2015 ▶). *In vitro* treatment with ABA increased intracellular levels of cAMP and evoked PPAR γ expression (Bassaganya-Riera et al., 2011 ▶; Bruzzone et al., 2008 ▶). Such activities were suppressed by blocking cAMP production or inhibiting PKA activity (Guri et al., 2010 ▶). It was also reported that ABA ability to suppress STZ-induced cognitive dysfunction is inhibited by PPARβ/δ and PKA antagonists (Khorasani et al., 2019 ▶). Therefore, it is plausible that ABA propensity on learning and memory, at least partially, might be accomplished by modulation of the cAMP/PKA signaling. 

 In addition to its significance in diabetes, PPARγ is involved in modulation of learning and memory performances and cognitive functions (Landreth et al., 2008 ▶; Lin et al., 2018 ▶). It was indicated that PPARγ agonist TZDs, improve memory performances in patients suffering from AD and in transgenic animal models of AD by improving glucose hemostasis and suppression of pro-inflammatory signaling in the brain (Cheng et al., 2016 ▶; Masciopinto et al., 2012 ▶). Moreover, oral administration of PPARγ agonist pioglitazone for six weeks, was shown to attenuate spatial memory deficiency in diabetic mice. The effects were accompanied by reductions of amyloid-beta (Aβ) proteins 40 and 42 levels in the hippocampus and cerebral cortex through inhibition of NF-κB pathway (Jiang et al., 2012 ▶). Therefore, these data suggested that ABA interaction with PPAR γ might be involved in its therapeutic benefits in learning and memory processes.

 In the present work, LTP was impaired in CA1 area of the hippocampus in response to STZ. STZ-induced diabetes model has been well established to investigate diabetes effects on cognitive performances in rodents (Yau et al., 2018 ▶; Yazir et al., 2019 ▶). STZ increased neuronal abnormalities in the hippocampus as revealed by induced microvascular damage and increased oxidative stress markers in rats (Yang et al., 2013 ▶). Moreover, STZ has been shown to disturb hippocampal synaptic plasticity in rats by down expression of BDNF and dysregulation of nitric oxide (Han et al., 2016 ▶). It was also reported that STZ inhibits adult neurogenesis and induces neuroinflammation in the rats brain (Bassani et al., 2018 ▶).

According to our results, ABA at the highest dose (20 µg/rat) was able to restore STZ-induced impairment of synaptic plasticity in CA1 area, in a manner that was blocked by PPARγ antagonist GW9662. However, because of the difficulties associated with electrophysiological procedure like test length, high cost, and animal mortality, as a limitation of this study, we were not able to test the other two groups treated with ABA (10 and 15 µg/rat). To the best of our knowledge, this work was the first to show ABA propensity on hippocampal synaptic plasticity. However, involvement of PPAR-γ signaling in synaptic transmission in the hippocampus is supported by previous studies (Bahrami et al., 2019 ▶). For instance, an *in vitro* trial indicated that PPARγ agonist ameliorated Aβ-mediated impairment of synaptic plasticity in CA1 area (Costello et al., 2005 ▶). In addition, a PPAR-γ agonist could restore cognitive deficiency and the reduce of dendritic spines in CA1 of obese rats (Sripetchwandee et al., 2014 ▶). So, ABA crosstalk with PPARγ signaling networks might be considered a potential explanation for ABA role in LTP induction. 

Collectively, the data indicated that central injection of phytohormone ABA could restore diabetes-induced learning and memory as well as synaptic plasticity destruction in rats predominantly via an interplay with PPARγ receptors. 
